# Oxidative stress-induced epigenetic changes associated with malignant transformation of human kidney epithelial cells

**DOI:** 10.18632/oncotarget.12091

**Published:** 2016-09-17

**Authors:** Prathap Kumar S. Mahalingaiah, Logeswari Ponnusamy, Kamaleshwar P Singh

**Affiliations:** ^1^ Department of Environmental Toxicology, The Institute of Environmental and Human Health (TIEHH), Texas Tech University, Lubbock, Texas, USA

**Keywords:** epigenetics, oxidative stress, DNA methylation, kidney cancer, histone modification

## Abstract

Renal Cell Carcinoma (RCC) in humans is positively influenced by oxidative stress status in kidneys. We recently reported that adaptive response to low level of chronic oxidative stress induces malignant transformation of immortalized human renal tubular epithelial cells. Epigenetic alterations in human RCC are well documented, but its role in oxidative stress-induced malignant transformation of kidney cells is not known. Therefore, the objective of this study was to evaluate the potential role of epigenetic changes in chronic oxidative stress-induced malignant transformation of HK-2, human renal tubular epithelial cells. The results revealed aberrant expression of epigenetic regulatory genes involved in DNA methylation (DNMT1, DNMT3a and MBD4) and histone modifications (HDAC1, HMT1 and HAT1) in HK-2 cells malignantly transformed by chronic oxidative stress. Additionally, both in vitro soft agar assay and in vivo nude mice study showing decreased tumorigenic potential of malignantly transformed HK-2 cells following treatment with DNA de-methylating agent 5-aza 2’ dC further confirmed the crucial role of DNA hypermethyaltion in oxidative stress-induced malignant transformation. Changes observed in global histone H3 acetylation (H3K9, H3K18, H3K27 and H3K14) and decrease in phospho-H2AX (Ser139) also suggest potential role of histone modifications in increased survival and malignant transformation of HK-2 cells by oxidative stress. In summary, the results of this study suggest that epigenetic reprogramming induced by low levels of oxidative stress act as driver for malignant transformation of kidney epithelial cells. Findings of this study are highly relevant in potential clinical application of epigenetic-based therapeutics for treatments of kidney cancers.

## INTRODUCTION

Renal cell carcinoma (RCC) originates from renal tubular epithelial cells and is the most common cancer of the genitourinary system, next to prostate and bladder cancer [1]. Although multiple risk factors are associated with RCC development, the most common feature among these risk factors is their ability to increase reactive oxygen species (ROS) generation and hence to induce oxidative stress. For example, well-established risk factors of RCC, such as smoking, obesity, hypertension, and chronic kidney diseases are known to increase oxidative stress burden in kidneys [2, 3]. Elevated but low levels of ROS act as key cell signaling molecules and can induce cell proliferation and transformation [4–6]. We recently reported that adaptive response to low level of chronic oxidative stress induces malignant transformation of immortalized human renal tubular epithelial cells potentially through acquisition of epithelial to mesenchymal transition (EMT) and stem cell characteristics [7].

Multiple mechanisms have been proposed for the role of oxidative stress in carcinogenesis. ROS regulates activity of cell signaling proteins involved in cell growth, proliferation as well as differentiation. For example, ROS activates proteins involved in cell proliferation such as MAPK (mitogen-activated protein kinase)/Erk1/2 (extra cellular regulated kinase 1/2) [8, 9]; cell survival/anti-apoptotic pathways such as PI3K/Akt signaling [10] and kinases such as PDK-1 (3- phosphoinositide-dependent kinase-1) [9]; and transcription factors involved in cell proliferation and cell cycle regulation such as NF-kβ [11]. Additionally, elevated levels of ROS causing oxidative stress has also been implicated in carcinogenesis through transcriptional regulation of genes. For example, upregulation of genes involved in cell cycle progression from G1 to S phase such as different Cyclins (*Cyclin D3, Cyclin B2 and, Cyclin E1*) [6], up-regulation of anti-apoptotic gene *Bcl-2* [12], increased expression of genes involved in cell motility and migration such as *MMPs* [13] and Snail, a key transcription factor regulating EMT and E-cadherin [14] by oxidative stress are known. ROS induced 8-hydroxy-2’ -deoxyguanosine (8-OHdG) DNA adduct may also lead to mutations and aberrant expression of genes. For example, oxidative DNA damage induced mutations leading to activation of oncogenes such as *Ras* or inactivation of tumor suppressor genes such as *p53* are also reported. However, the precise mechanism through which the ROS controls the transcriptional regulation of genes is not clear.

Recent studies suggest that epigenetic changes of DNA methylation and histone modifications play an important role in regulation of gene expression at the transcript level. For example epigenetic regulation of genes involved in cell cycle regulation [15], cell survival [16], apoptosis [17, 18], DNA repair [19, 20], EMT [21] and stem cell characteristics [22] are reported in different cell models. In addition, epigenetic changes may also contribute to increased DNA mutations. For example, DNA hypermethylation-mediated silencing of *MGMT* (*O^6^-methylguanine-DNA methyl transferase*) gene, which encodes for protein involved in DNA repair, increases mutations in oncogenes and tumor suppressor genes to promote carcinogenesis [20, 23]. Therefore, aberrant expression of genes through epigenetic mechanism may eventually lead to altered cellular signaling, impaired DNA damage repair, increased mutations, genomic instability and consequently predispose cells to malignant transformation.

Accumulating evidence also suggests potential role of epigenetic changes in the development and progression of RCC [24–27]. Relatively lower frequency of mutations in RCC as compared to other cancers and consistent reports of DNA hypermethylation mediated silencing of several tumor suppressor genes, further suggest greater role of epigenetic changes rather than genetic changes in renal carcinogenesis [24]. For example, increased DNA methylation was reported in histologically non-cancerous renal parenchyma collected from renal cancer patients, when compared to corresponding normal renal tissue collected from patients without any primary RCC [28]. Hypermethylation mediated silencing of tumor suppressor genes such as *p16, E-cadherin* [29, 30], *VHL* [31, 32] and *RASSF1A* [33] [34] have been reported in both renal cell carcinoma cell lines as well as in RCC patients. Similarly, multiple aberrant post-translational modifications in histones, such as, H3K18Ac and H3K4 methylation have also been implicated in renal cancer development and progression [35, 36].

These reports clearly suggest that in addition to genetic changes such as mutations, epigenetic changes of DNA methylation and histone modifications also play an important role in renal cancer development. However, the role of epigenetic changes in chronic oxidative stress-induced renal carcinogenesis is not known. Epigenetic modifications are potentially reversible, and hence understanding of epigenetic regulation during malignant transformation of renal epithelial cells will be of great value to develop new strategies to prevent as well as to treat RCC at early stages. Therefore, the objective of this study was to evaluate the epigenetic changes that occur during oxidative stress-induced malignant transformation of renal tubular epithelial cells.

## RESULTS

In this study, HK-2 human kidney epithelial cells malignantly transformed by chronic exposure to oxidative stress were used to evaluate the role of epigenetic changes in oxidative stress-induced carcinogenesis. HK-2 cells are immortalized but non-tumorigenic normal human kidney tubular epithelial cells. We have recently reported that chronic exposure (6 months) to low level of oxidative stress results in malignant transformation of HK-2 cells as confirmed by both in vitro and in vivo tumorigenicity assays, whereas the chronic exposure to relatively high level of oxidative stress results in significant adaptation to oxidative stress-induced cytotoxicity [Mahalingaiah et al., 2015]. We named the oxidative stress-induced malignantly transformed HK-2 cells as OT-HK-2 cells, and HK-2 cells adapted to high level of oxidative stress as OA-HK-2 cells. Using these two cell models, the role of epigenetic changes in oxidative stress-induced carcinogenesis was evaluated by measuring the expression of epigenetic regulatory genes at the transcript and protein levels. The oxidative stress-induced epigenetic changes in histone acetylation and methylation were also measured by Western blot analysis. To determine the role of DNA hypermethylation in oxidative stress-induced malignant transformation, the OT-HK-2 cells were treated with DNA demethylating agent 5-aza 2’ dC and its effects on cell growth, anchorage independent growth and in vivo tumorigenic potential of these cells were evaluated. Results of these analyses are presented in the following sections.

### Transcript level changes in expression of genes involved in DNA methylation

**Figure 1:**
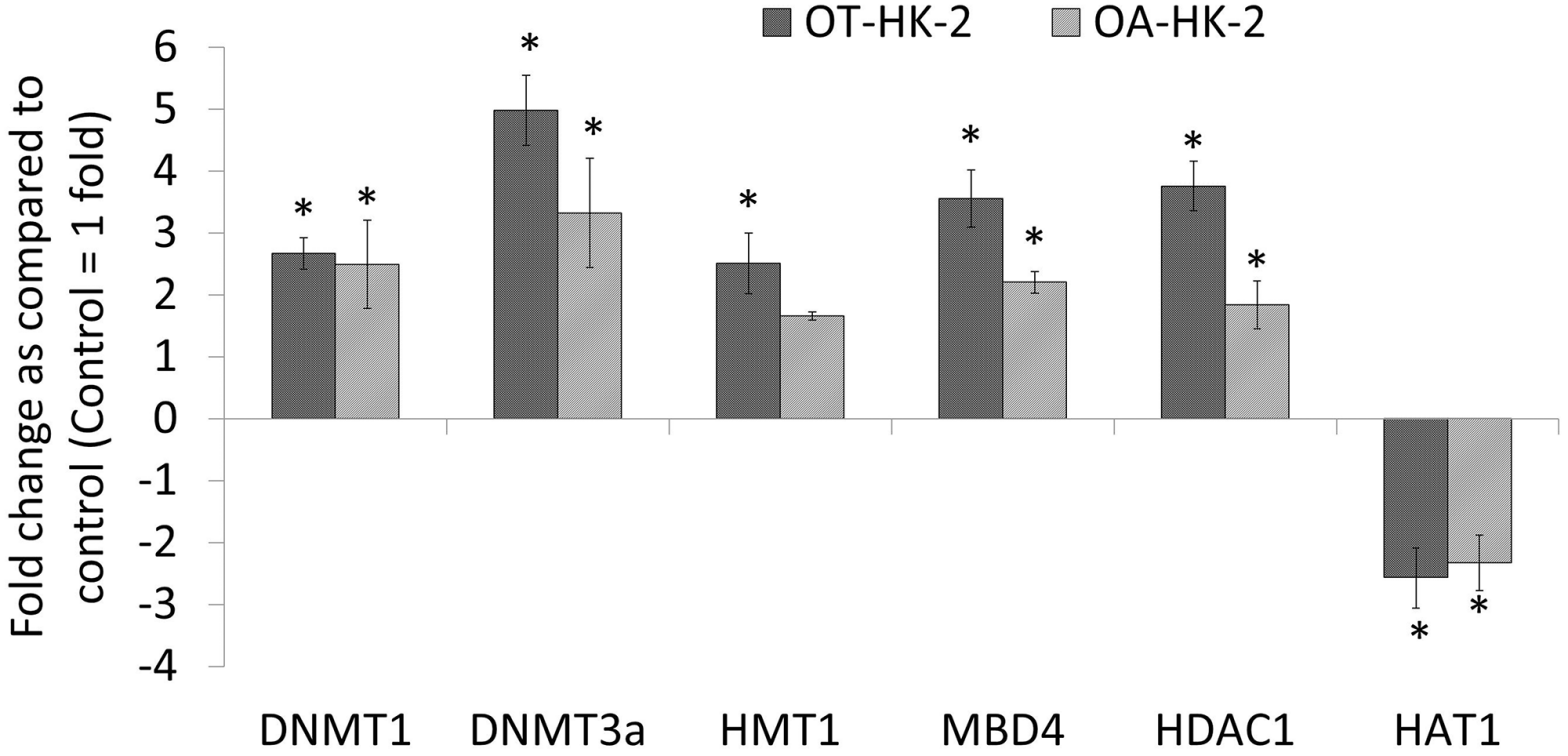
Histogram showing expression of epigenetic regulatory genes in HK-2 cells exposed to chronic oxidative stress Total RNA isolated from HK-2, OT-HK-2 and OA-HK-2 cells was used to perform one-step real-time quantitative reverse transcription PCR as described in materials and methods. Cycle threshold value (Ct value) of each gene was normalized to the Ct value of housekeeping gene GAPDH from the same sample. The gene expression in fold change was calculated and histogram was plotted using the means of triplicate values. The error bars represent the standard deviation of the mean (±SD). An *asterisk* (*) indicates statistically significant (p<0.05) change, when compared to control.

Changes in expression of genes involved in maintenance of DNA methylation (*DNMT1*), de novo DNA methylation (*DNMT3a*), and methyl DNA binding protein (*MBD4*) were evaluated in HK-2 cells exposed to chronic oxidative stress by quantitative real time PCR. Statistically significant increase in *DNMT1*, *DNMT3a* and *MBD4* expression was observed in both OT-HK-2 and OA-HK-2 cells. In OT-HK-2 cells, increase by 2.8 folds, 4.9 folds and 3.5 folds were observed in *DNMT1, DNMT3a* and *MBD4* respectively (Figure 1). Similarly, in OA-HK-2 cells, an increase by 2.4 folds, 3.3 folds and 2.2 folds in the expression of *DNMT1, DNMT3a* and *MBD4* gene transcripts respectively were observed (Figure 1).

### Changes in expression of histone modifying genes transcripts

Effect of chronic oxidative stress on histone modification was evaluated by analyzing changes in expression of representative genes involved in transfer of methyl groups to histones (*HMT1*), transfer of acetyl groups to histones (*HAT1*) and removal of acetyl groups from histone tail (*HDAC1*). Significant increase in *HDAC1* expression and significant decrease in *HAT1* expression was observed in both OT-HK-2 and OA-HK-2 cells. *HDAC1* expression was increased by 3.7 folds in OT-HK-2 cells and by 1.8 folds in OA-HK-2 cells. Similarly, expression of *HAT1* was decreased by 2.5 and 2.3 folds in OT-HK-2 and OA-HK-2 cells, respectively (Figure 1). Significant increase in expression of *HMT1* by 2.5 fold was observed only in OT-HK-2 cells.

### Changes in expression of epigenetic regulatory proteins and histone modifications

**Figure 2:**
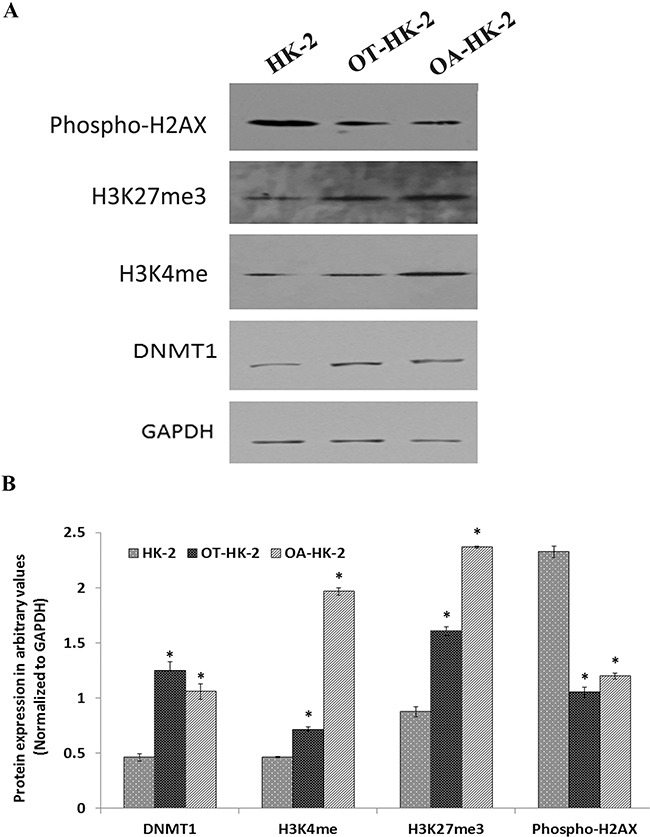
Representative Western blot images A. and their relative band intensity histograms B. of epigenetic regulatory proteins involved in DNA methylation and histone modifications (methylation) Signal intensity of bands was measured (in triplicates) by Image J software and each value was normalized to GAPDH signal intensity. The error bars represent the standard deviation of the mean (±SD). Symbol *asterisk* (*) indicates statistically significant (p<0.05) change, when compared to control.

**Figure 3:**
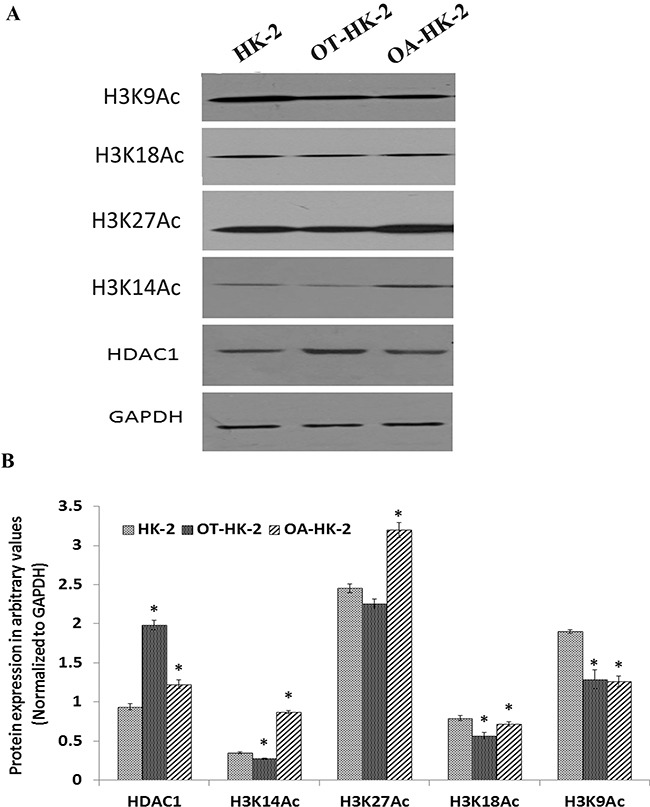
Representative Western blot images A. and their relative band intensity histograms B. of epigenetic regulatory proteins involved in histone acetylation and histone modifications (acetylation) Signal intensity of bands was measured (in triplicates) by Image J software and each value was normalized to GAPDH signal intensity. The error bars represent the standard deviation of the mean (±SD). Symbol *asterisk* (*) indicates statistically significant (p<0.05) change, when compared to control.

Western blot analysis was performed to further confirm the transcript level epigenetic regulatory gene expression changes and to evaluate histone modifications in HK-2 cells adapted to chronic oxidative stress. Results of the Western blot revealed a significant increase in DNMT1 expression in malignantly transformed HK-2 cells (OT-HK-2 cells) as well as in HK-2 cells adapted to cytotoxic effect of high level of oxidative stress (OA-HK-2 cells) as compared to control cells. This increase in DNMT1 expression was greater in OT-HK-2 cells when compared to OA-HK-2 cells (Figure 2). Similarly, significant upregulation of HDAC1 was evident in OT-HK-2 and OA-HK-2 cells (Figure 1). Increased HDAC1 expression was also associated with significant decrease in histone H3 acetylation (H3K14 and H3K9) in OT-HK-2 cells (Figure 3). In OA-HK-2 cells, significant decrease in H3K9 acetylation was observed, whereas there was no significant change in acetylation level of H3K14. There was no significant change in acetylation level of H3K18 in both OT-HK-2 and OA-HK-2 cells. But significant increase in level of H3K27 acetylation was observed in OA-HK-2 cells. Significant increase in histone H3 methylation (H3K27me3 and H3K4me3) was observed in both OT-HK-2 and OA-HK-2 cells (Figure 2). In addition, significant down-regulation of phospho-H2AX level was evident in both OT-HK-2 and OA-HK-2 cells (Figure 2).

### Effect of non-cytotoxic dose of 5-aza 2’ dC on growth of HK-2 cells adapted to chronic oxidative stress

**Figure 4:**
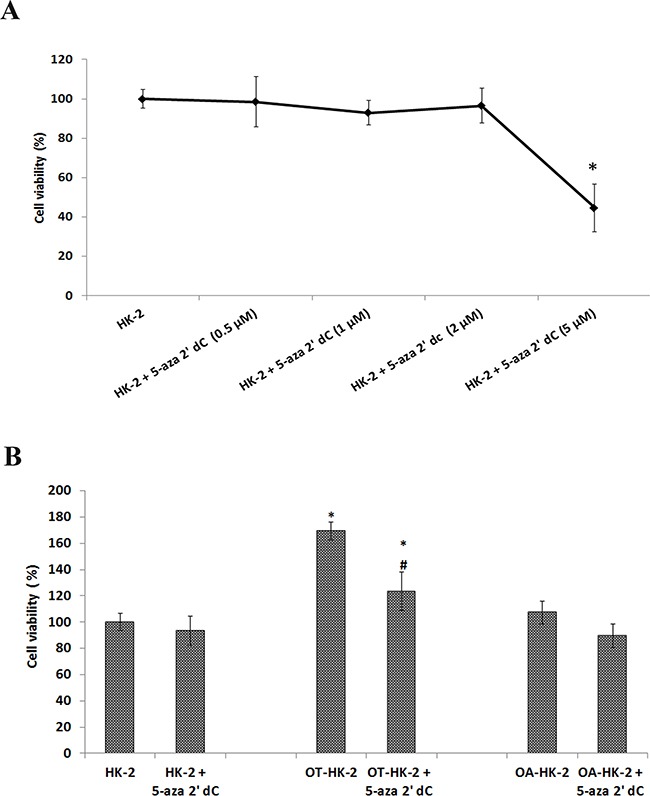
A. Line graph showing effect of 5-aza 2’ dC on cell viability of normal HK-2 cells as evaluated by MTT assay Control HK-2 cells were exposed to increasing concentrations of 5-aza 2’ dC to choose highest non-cytotoxic level to use for DNA demethylation. **B**. Histogram showing the effects of 5-aza 2’ dc on cell growth of HK-2 cells exposed to chronic oxidative stress. MTT data were converted into percentage of control (Control = 100%). The error bars represent the standard deviation of the mean (±SD). An *asterisk* (*) indicates statistically significant (p<0.05) change, when compared to vehicle control. Symbol *hash* (#) indicates statistically significant (p<0.05) change, when compared to respective group without 5-aza 2’ dC treatments.

To optimize the dose of 5-aza 2’ dC that can induce demethylation without any cytotoxic effect, HK-2 cells were treated with a range of 5-aza 2’ dC from 0.5μM to 5μM and cell viability was assessed by MTT assay. Results of MTT assay showed no significant effect of 5-aza 2’ dC treatments up to 2μM for 48 hours on cell growth/viability of normal HK-2 cells (Figure 4A). Significant cytotoxicity with 55.4% decrease in cell growth was observed in HK-2 cells treated with 5μM 5-aza 2’ dC for 48 hours. Based on these results, the highest dose of 5-aza 2’ dC (2μM) with no significant effect on cell viability of normal HK-2 cells was selected to induce DNA demethylation in OT-HK-2 and OA-HK-2 cells.

Using the 2μM non-toxic dose of 5-aza 2’ dC, cells were further treated and its effect on growth and tumorigenic potential of OT-HK-2 and OA-HK-2 cells were determined. Result of MTT assay confirmed significant increase in growth of malignantly transformed HK-2 cells (OT-HK-2), compared to passage matched control. About 69.4% increase in cell growth was observed in OT-HK-2 cells (Figure 4B). Following 5-aza 2’ dC treatment, 27.2% decrease in cell growth was observed in OT-HK-2 cells, when compared to same group of cells without 5-aza 2’ dC treatment. In HK-2 cells adapted to high level of oxidative stress (OA-HK-2 cells), 16.6% decreases in cell growth was observed with 5-aza 2’ dC treatment, but this change was not statistically significant. Results of MTT assay, confirmed significant decrease in growth of malignantly transformed HK-2 cells (OT-HK-2 cells) with 5-aza 2’ dC treatment.

### Effect of 5-aza 2’ dC on in vitro tumorigenic potential of malignantly transformed HK-2 cells

**Figure 5:**
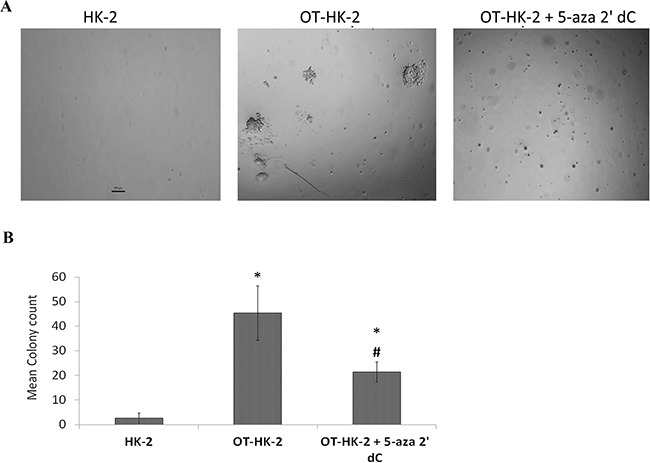
Representative images (40X magnification) of soft agar colonies A. and histogram of colony count B. showing effect of 5-aza 2’ dc treatments on in vitro tumorigenic potential of HK-2 cells exposed to chronic oxidative stress OT-HK-2 cells were harvested, plated, and allowed to grow in soft agar as described in materials and methods section and observed microscopically for colony formation and images were captured on Day 14.

To further evaluate the effect of demethylation on anchorage-independent growth and colony formation (in vitro tumorigenic potential) of malignantly transformed HK-2 cells (OT-HK-2 cells), soft agar assay was performed. Anchorage-independent growth and colony formation on soft agar was observed in OT-HK-2 (Figure 5), whereas 5-aza 2’ dC treatment significantly decreased both size and number of soft agar colonies formed by OT-HK-2 cells. Hence the results of the soft agar assay confirmed that 5-aza 2’ dC treatment decreased in vitro tumorigenic potential of malignantly transformed HK-2 cells.

### Effect of 5-aza 2’ dC on in vivo tumorigenic potential of malignantly transformed HK-2 cells

**Figure 6:**
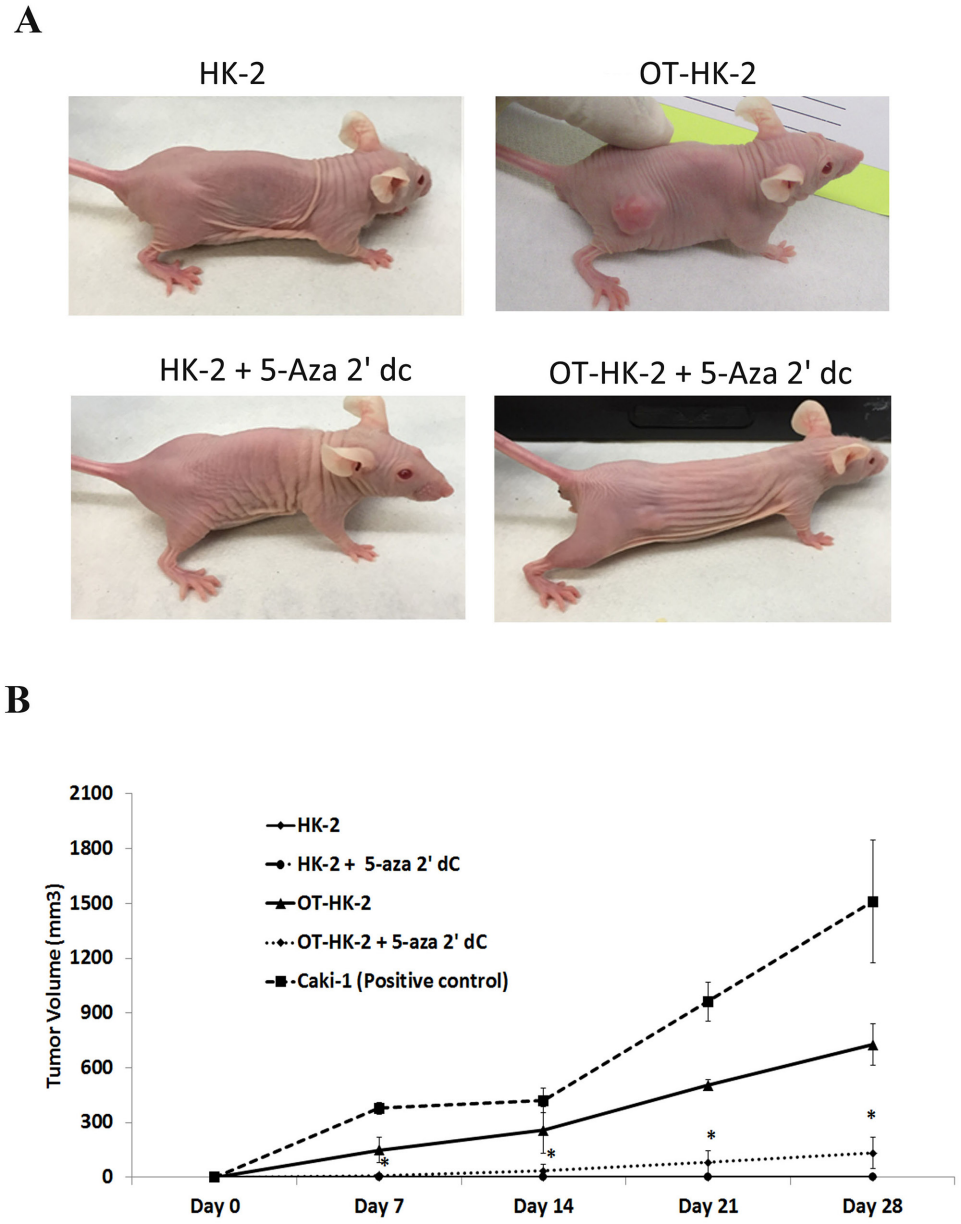
Representative images showing effect of 5-aza 2’ dc on in vivo tumor formation potential of malignantly transformed HK-2 cells OT-HK-2 cells and passage matched control HK-2 cells were treated with 5-aza 2’ dc and inoculated subcutaneously into right flank of athymic nude female mice and observed for tumor formation as described in material and method section. All animals were observed for tumor formation daily for 4 weeks. Tumor volume (mm^3^) was measured in weekly intervals in all the tumor-bearing animals and presented in the graph in comparison to positive control renal cancer cells (Caki-1). In animals injected with HK-2 and HK-2 + 5-aza 2’ dc treated cells, there was no visible tumor formation at the site of injection. Hence tumor volume of “0” is used for both these groups and lines are overlapping on the x-axis of the graph. Each data point represents mean tumor volume ± standard deviation (SD). An *asterisk* (*) indicates statistically significant (p<0.05) change, when compared to OT-HK-2 cells inoculated animals at respective intervals.

**Figure 7:**
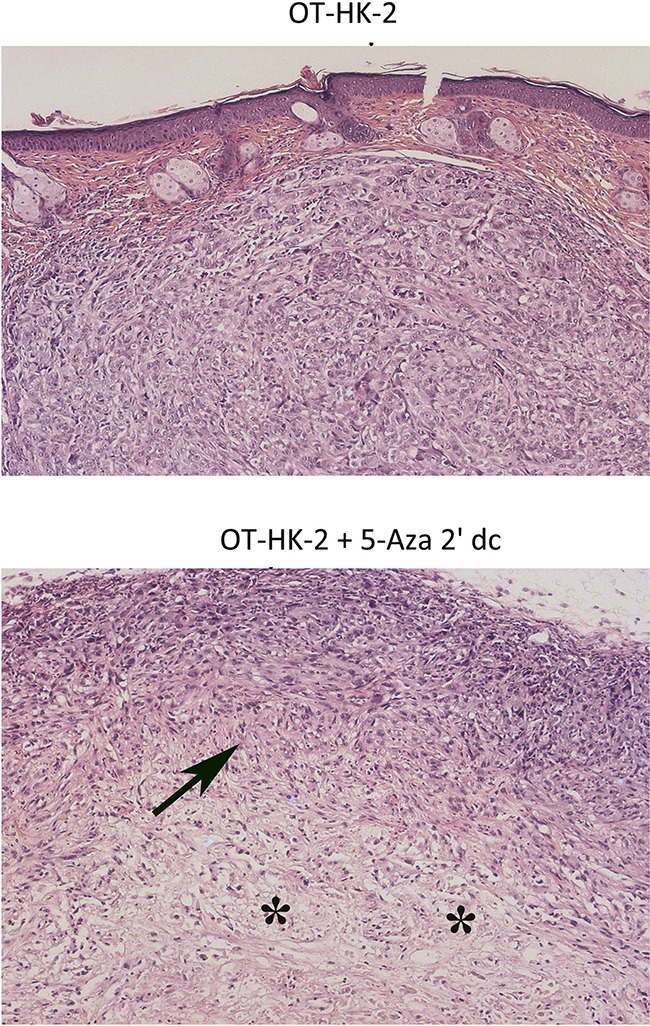
Representative photomicrographs showing histopathological changes in xenograft tumor samples Tumor samples from athymic nude mice inoculated with OT-HK-2 cells with and without 5-aza 2’ dc treatment were fixed in 10% neutral buffered formalin, processed for paraffin embedding, sections (5μM) were cut, stained with haemotoxylin and eosin stain and evaluated microscopically for histopathological changes. In 5-aza 2’ dc treated group, decreased tumor volume/growth was histologically characterized by areas of decrease in cellularity (arrows) with prominence of connective tissue portion (asterisk).

To evaluate the effect of 5-aza 2’ dC treatment on *in vivo* tumorigenic potential, subcutaneous xenograft study was performed in Athymic nude mice. Visible tumor formation in subcutaneous flank region was observed in 3 mice out of 5 inoculated with HK-2 cells chronically exposed to lower level of oxidative stress (OT-HK-2 cells), confirming their malignant transformation. In mice inoculated with OT-HK-2 cells treated with 5-aza 2’ dC for 48 hours, only 2 mice out of 5 developed visible tumor formation. There was no visible tumor growth at the site of injection in remaining 3 animals. In addition, significant decrease in visible tumor size and tumor volume at all-time points evaluated (Day 7, Day 14, Day 21 and Day 28), was observed in mice inoculated with 5-aza 2’ dC treated OT-HK-2 cells (Figure 6). Decrease in tumor growth with hypo-cellularity as a result of 5-aza 2’ dC treatment was also confirmed by histological examination of tumor samples (Figure 7). Tumor formation was observed in all the mice inoculated with well-established renal cell carcinoma cells (Caki-1 cell line), which are used as a positive control cells for xenograft study. Hence results of xenograft study further confirmed 5-aza 2’ dC induced decrease in tumorigenic potential of malignantly transformed HK-2 cells.

## DISCUSSION

Oxidative stress has been associated with the etiology of multiple types of cancer including renal cell carcinoma. We recently reported that chronic exposure to oxidative stress leads to malignant transformation of HK-2 cells, an immortalized cell line derived from human normal kidney tubular epithelium. Transformation of normal cells into malignant cells is a multistage process involving clonal selection of cells harboring either inherited or acquired genetic and epigenetic changes contributing to uncontrolled growth advantage [37]. Genetic changes such as alterations in cell signaling pathways and oxidative DNA damage are commonly associated with oxidative stress-induced malignancy [38]. Even though multiple reports exist to support the oxidative stress-induced epigenetic changes, there is no direct evidence to implicate oxidative stress-induced epigenetic changes in malignant transformation of human kidney epithelial cells. Therefore, the objective of this study was to evaluate the significance of chronic oxidative stress-induced epigenetic changes in malignant transformation of HK-2 cells. Results of our study showing significant alterations in epigenetic regulatory proteins as well as decrease in both in vitro and in vivo tumorigenicity of malignantly transformed cells following treatment with DNA de-methylating agent (5-aza 2’ dC) suggested that epigenetic reprogramming play an important role in oxidative stress-induced malignant transformation of kidney epithelial cells. To our knowledge, this is the first report that provides direct evidence for oxidative stress-induced epigenetic alterations as driver for malignant transformation of normal kidney epithelial cells.

Among different types of epigenetic modifications, DNA methylation has been considered as an important regulatory mechanism in carcinogenesis. Alterations in DNA methylation pattern and expression of DNA methylation regulatory proteins have been widely implicated in renal carcinogenesis [39, 40]. Except for VHL gene, the occurrences of mutations are relatively infrequent in RCC. On the other hand numerous reports of DNA hypermethylation-mediated silencing of several tumor suppressor genes further suggest the important role of epigenetic changes in renal carcinogenesis [24]. A significant increase in expression of DNMTs (DNMT1 and DNMT3a) as observed in OT-HK-2 and OA-HK-2 cells in this study suggests a potential role of DNA hypermethylation in chronic oxidative stress-induced malignant transformation of HK-2 cells. Moreover, a decrease in growth and tumorigenic potential of malignantly transformed HK-2 cells following 5-aza 2’ dC treatment in our study further support a role of DNA hypermethylation in chronic oxidative stress-induced renal carcinogenesis. Similar results of DNA hypermethylation during oxidative stress-induced anchorage independent growth and malignant transformation of melanocytes have been reported [41]. ROS-induced DNA hypermethylation have also been suggested to be associated with colorectal cancer [42], hepatic cancer [43] and pancreatic cancer [44]. Oxidative stress may influence DNA methylation through different mechanisms including upregulation of DNA methyl transferase enzymes (DNMTs), altered structure and function of epigenetic regulatory proteins such as DNMTs and/or by recruitment of epigenetic regulatory proteins, such as polycomb proteins, at the sites of oxidative stress-induced DNA modifications/damage [45, 46]. Increased expression of DNMTs and their correlation to increased aggressiveness and poor prognosis in patients have been reported in multiple cancers including breast, colon, prostate, lung, and kidney cancers [47]. Therefore, finding of this study together with previous reports suggest that chronic oxidative stress, through increased expression of DNMTs, leads to DNA hypermethylation-mediated inactivation of tumor suppressor genes and consequently the carcinogenesis.

Participation in repressive complex of proteins is yet another known mechanisms through which DNMTs can function in transcriptional silencing of genes. For example, increased recruitment of DNMT1 to the sites of oxidative damaged chromatin in hydrogen peroxide exposed cells resulting in formation of a large transcriptionally repressive complex containing DNMT1, DNMT3b and polycomb repressive complex 4 (PRC4) components including SIRT have been proposed as yet another mechanism for oxidative stress-mediated DNA hypermethylation and transcriptional silencing of genes [48]. Whether the increased expression of DNMT1 in cells chronically exposed to oxidative stress as observed in the present study contributed to increased cell survival through suppressing the expression of apoptotic genes through similar mechanism involving transcriptionally repressive complex is a question that needs to be addressed in future study.

In addition to increase in DNMTs expression, the data of this study also revealed a significant increase in MBD4 expression in renal epithelial cells malignantly transformed during adaptation to chronic oxidative stress. Methyl-CpG-binding domain (MBDs) proteins play an important role in DNA methylation-mediated silencing of genes and in integrating both DNA methylation and histone modifications. MBD4 is an essential protein which promotes cell survival during oxidative stress [49]. Synergistic cooperation of MBD4 in DNMT1 mediated hypermethylation and transcriptional repression of genes to protect cells against oxidative stress-induced cell death has been reported in human primary and transformed cells [49]. Increased level of both MBD4 and DNMT1 have been reported in mammalian cells exposed to oxidative stress [49] and it has been concluded that both these proteins are recruited to sites of oxidative DNA damage and they synergistically interact to induce transcriptional repression of genes such as CDKN1A/p21 (DNA damage response gene) and MSH4. Multiple reports confirm that, deficiency of MBD4 increases sensitivity of human cells to DNA damage by H_2_O_2_ induced oxidative stress [49]. Therefore, in addition to increased expression of DNMT1, the increased level of MBD4 as observed in this study can also potentially impact the increased cell survival through chromatin remodeling at the site of oxidative DNA damage resulting in loss of DNA damage-dependent apoptotic response.

In addition to DNA methylation, post-translational modifications in histone tails also play an important role as an additional layer of epigenetic regulation [50]. In the present study, a significant increase in expression of HDAC1, an enzyme involved in post-translational modification of histones was also observed in malignantly transformed OT-HK-2 cells. Oxidative stress-induced histone modifications through altered structure and function of HMTs and HDACs as well as recruitment of histone modulating protein complexes to the sites of H_2_O_2_-induced DNA damage involving promoter regions of tumor suppressor genes may contribute to malignant transformation of cells [48]. HDACs play a role in normal development of kidney as well as in pathogenesis of kidney diseases including cancer through influencing expression of several genes involved in carcinogenesis [51]. It has been reported that HDAC1 is critical for mitosis in cancer cells and its absence induces activation of caspases leading to apoptosis mediated cell death [52]. HDAC mediated regulation of cell proliferation and apoptosis may also result from deacetylation of non-histone proteins such as transcription factors and proteins involved in regulation of cell proliferation and apoptosis [51, 53]. HDAC1 mediated cell proliferation regulation is mainly mediated by suppression of key target gene p21, a cyclin dependent kinase inhibitor and an important growth suppressive gene [54–57]. Treatment of renal cell carcinoma cell lines with HDAC inhibitor, VPA results in increased expression of p21with decreased proliferation of cells [58]. HDAC mediated transcriptional regulation is also mediated by association of HDAC proteins with multi protein corepressor complex or through direct interaction of HDAC proteins with transcription factor of different genes such as p53, Rb, NF-kβ [59]. Role of HDAC1 in regulation of oxidative stress mediated apoptosis has been mainly implicated for its association with p53 protein [60, 61]. Recent study also demonstrated that HDAC1-induced decreased expression of thioredoxin binding protein-2 will result in decreased sensitivity of cancer cells to oxidative stress induced cytotoxicity (Takuya Kato et al 2009). Another study revealed that increased oxidative stress in kidney cells increases HDAC level and decrease acetylation of proteins, and therefore this study further suggest the impact of oxidative stress on the function of histone deacetylase [62]. Therefore, these previous reports and the finding of increased expression of HDAC1 in H_2_O_2_ exposed kidney epithelial cells in this study, together suggest that adaptive response of cells to oxidative stress-induced cytotoxicity leading to increased cell survival is potentially mediated by HDAC1-mediated histone modifications and chromatin remodeling at the target genes for cell survival. Future studies for identification of these target genes would further help in mechanistic understanding of the epigenetic basis for oxidative stress-induced carcinogenesis.

Cross talk between DNA methylation and histone modifications in cancer cells is also supported by enrichment of specific histone marks in the regions of DNA hypermethylation [21]. DNA methylation-induced gene silencing is commonly associated with decreased acetylation of histone H3 and H4 proteins [63]. Synergistic epigenetic reactivation of gene expression following combination of DNA de-methylating agent (5-aza 2’ dC) and histone modifying drugs (Trichostatin), also support these reports [64]. Interaction between MBD proteins and HDAC proteins also play a role in regulating cross talk between DNA methylation and histone acetylation [65]. Reports showing influence of HDAC inhibitors on DNA methylation [66] and DNMT inhibitors on histone modifications [67], further confirms cross talk between DNA methylation and histone post-translational modifications. Histone H3 and H4 commonly undergo different type of post-translational modifications that determine their association with either transcriptionally active or repressive chromatin. Acetylation of histone H3 is considered as a well characterized and key epigenetic modification involved in regulating expression of genes involved in carcinogenesis [68, 69]. Data from this study also revealed that the increase in HDAC1 expression during oxidative stress-induced transformation of HK-2 cells is also associated with decrease in global histone H3 acetylation involving H3K9, H3K18, H3K27 and H3K14. Recent report also suggest that epigenetic changes of global histone modifications, such as acetylation of H3K9 and H3K18, are decreased with progression of RCC and hence these histone modifications can be used as potential markers for RCC prognosis [35]. Oxidative stress-induced hypo acetylation of histones is reported at both global as well as gene-specific promoter levels. H_2_O_2_-induced transcriptional repression of p21 is associated with formation of hypo-acetylated repressive chromatin at the p21 promoter region of human breast cancer cell lines [70]. Increased cytotoxic effect of mTOR inhibitors in combination with HDAC inhibitors on RCC xenograft growth are also reported in pre-clinical studies [71]. Decrease in acetylation of Histone H3K16 along with activity of MYST (Moz-Ybf2/Sas3-Sas2-Tip60), an enzyme involved in transfer of acetyl group to H3K16 has been correlated with pathogenesis of RCC [72]. Inverse correlation between acetylation of histone H3 and progression/metastasis of renal cell carcinoma has been reported [72].

Similar to histone H3 acetylation, histone H3 methylation is implicated in cancer development. Histone methylation is mediated by group of enzymes called histone methyl transferases (HMT), which can transfer methyl groups to arginine or lysine to induce mono, di or tri methylation of histone proteins. Significant increase in HMT1 expression and histone H3K27 tri-methylation and H3K4 methylation levels in our cell model further indicates a potential role of histone methylation in adaptive response to chronic oxidative stress. Changes in methylation involving H4K9 and H3K27 are commonly reported in cancer cells [73]. Histone H3K27 tri-methylation represent repressive chromatin with loss of tumor suppressor gene expression may eventually lead to malignant transformation of cells [74, 75]. Increased levels of global histone H3K27 and H3K4 methylation have been reported in RCC patients and also their expressions were correlated with progression and recurrence of RCC [36, 76]. H_2_O_2_-induced global histone modifications with increase in histone H3 methylation (H3K27 and H3K4) and decrease in acetylation of histone H3K9 reported in immortalized bronchial epithelial cells [77] further support our study findings. Oxidative stress-induced histone H3K4 methylation has been related to increased oxidation of Fe (II) to Fe (III) resulting in decreased activity of histone modifying enzymes such as JmjC domain-containing histone demethylases [77]. Therefore, these previous reports and the finding of increased global histone H3 modifications observed during oxidative stress-induced malignant transformation of HK-2 cells in this study further support the role of histone modifications in oxidative stress-induced kidney carcinogenesis either directly or indirectly through epigenetic protein complex-dependent chromatin remodeling and changes in genes expression.

Histone H2AX, a variant of H2A protein play an important role as a guardian of the genome, and loss of H2AX may induce genomic instability with increased chance of cancer development. Dynamic nucleosome remodeling including phosphorylation of H2AX protein by DNA damage responsive kinases play a key role in oxidative stress-induced DNA repair mechanism [48, 78]. Phosphorylation of H2AX, specifically facilitate stable interaction between base excision repair (BER) proteins and sites of damaged DNA induced by oxidative stress [79]. In the present study, a significant decrease in phospho-H2AX (Ser139) level was observed in HK-2 cells malignantly transformed by chronic oxidative stress (OT-HK2 cells) Recently it has been reported that phosphorylation of H2AX at serine 139 and serine 16 positions can prevent malignant transformation of cells [80]. Hence, the result of our study showing a decrease in the level of phospho-H2AX (Ser139) is consistent with this previous report and further supports the role of decreased level of phospho-H2AX in increased cell survival and malignant transformation. Increased formation of phospho-H2AX observed in human lung carcinoma cells exposed to ionizing radiation [81] and decreased levels of phospho-H2AX with increased expression of DNMT1 by histone deacetylase (HDAC) inhibitors [82, 83] further support our results in malignantly transformed renal epithelial cells.

To our knowledge, this is the first report providing direct evidence for the role of epigenetic mechanism as driver for the chronic oxidative stress-induced malignant transformation of renal tubular epithelial cells. Further study on identification and functional characterization of genes affected by oxidative stress-induced epigenetic alterations could potentially help in early detection and epigenetic therapy of renal cancers.

## MATERIALS AND METHODS

### Chemicals

Hydrogen peroxide (H_2_O_2_) 30% (w/v) solution, 3-(4,5 dimethylthiazol-2-yl)-2,5-diphenyltetrazolium bromide (MTT), and 5-Aza-2’-deoxycytidine (5-aza 2’ dC) were purchased from Sigma (St. Louis, MO). Serum free Keratinocyte medium with growth factors, trypsin/EDTA solution, and Trizol reagent were purchased from Invitrogen Inc. For protein extraction, RIPA lysis buffer (1X) was purchased from Santa Cruz Biotechnology, Inc.

### Cell culture and treatments

HK-2 cells were purchased from ATCC and maintained in Keratinocyte serum free medium supplemented with human recombinant EGF, bovine pituitary extract and 1% antibiotic-antimycotic solution. HK-2 cells are immortalized human proximal tubular epithelial cells derived from normal kidney and are non-tumorigenic. HK-2 cell cultures were grown and maintained at 37°C with a humidified atmosphere containing 5% CO_2_. H_2_O_2_is known to increase intracellular levels of highly reactive hydroxyl radical and has been used in multiple studies to induce oxidative stress in both in vitro cell cultures as well as in animal models [84–86]. Therefore, for chronic exposure of cells to oxidative stress, actively growing HK-2 cells were treated with 25μM (a lower non-cytotoxic) and 250μM (a higher cytotoxic) concentrations of H_2_O_2_. These cells, after reaching a confluency of about 80%, were sub-cultured, grown for 24 hours, and then given fresh treatment of H_2_O_2_. Using this method, cells were grown for 6 months in H_2_O_2_ containing media. HK-2 cell cultures for chronic treatment were maintained and treated in triplicate and also parallel cultures were grown and maintained as a passage matched controls. To evaluate the role of DNA hypermethylation in oxidative stress- induced transformation and adaptation to stress, HK-2 cells exposed to chronic oxidative stress were treated with DNA de-methylating agent (2μM of 5-aza 2’ dC) for 48 hours and then used for analysis of cell growth and tumorigenic potential. Control group HK-2 cells were treated with vehicle (0.001% DMSO) only in the culture medium.

### Cell viability assay

HK-2 cells exposed to chronic oxidative stress and passage matched control cells were seeded at a density of 2000 cells per well in 96 well flat bottom culture plates and allowed to grow for 24 hours at 37°C with 5% CO_2_ in an incubator. To evaluate the role of DNA methylation, respective groups of cells were given exposure (48 hours) to 5-aza 2’ dC as mentioned earlier, and then cells were incubated with 10% MTT solution for 3 hours at 37°C with 5% CO_2_. Cell culture media was removed and DMSO (150μL) was added in to each well to dissolve water insoluble formazan crystals formed in mitochondria. The intensity of the color formed due to DMSO dissolved formazan in each well was measured at 570 and 630 nm absorbance using microplate reader. Treatments were performed in triplicates and experiment was repeated twice.

### Soft agar assay for colony formation

Soft agar colony formation assay was performed to evaluate the effect of 5-aza 2’ dC on anchorage-independent growth (in vitro assay to confirm malignant transformation) of HK-2 cells with chronic exposure to oxidative stress. Base layer of soft agar was prepared by pouring 1.5 mL of 0.5% agar in Keratinocyte media in each well of 6 well plates and allowed to polymerize for few minutes. Top layer of soft agar was prepared using 0.35% agar in Keratinocyte media and cells from each treatment groups were mixed (5000 cells in 1.5 mL soft agar solution) and plated over the base layer in each well. All the soft agar plates were allowed to solidify and then maintained at 37°C with a humidified atmosphere containing 5% CO_2_. Colony formation of cells on soft agar was monitored by microscopic observation on daily basis. On Day 14, colonies were counted manually using microscope and representative images were taken. The soft agar grown colony numbers in treatment groups were expressed as a percentage compared to vehicle control.

### Evaluation of in vivo tumorigenic potential of transformed HK-2 cells

Effect of DNA demethylation by 5-aza 2’ dC treatments on in vivo tumorigenic potential of HK-2 cells malignantly transformed by chronic oxidative stress was evaluated by subcutaneous xenograft study in athymic nude mice. A total of 25 athymic nude female mice (5-6 week old) were purchased from Charles River Laboratories and were allowed for acclimatization for 7 days. These mice were randomly divided into five groups with 5 mice in each group. Group 1 and group 2 animals were injected with HK-2 cells exposed to vehicle control and 5-aza 2’ dC, respectively. Group 3 and group 4 animals were injected with transformed HK-2 cells (OT-HK-2 cells) exposed to vehicle control and 5-aza 2’ dC, respectively. Group 5 animals were injected with Caki-1 renal carcinoma cells as a positive control. Mice in respective group were subcutaneously (right flank region) inoculated with 10^7^ cells suspended in 200μL solution of 1X phosphate buffered saline. All the animals were maintained by providing standard laboratory conditions in individually ventialted (IVC) cages with *ad libitum* food and water. Animals were subjected for daily observation for their general health condition and also for tumor fromation. Tumor formed at the site of injection was measured at weekly intervals for 4 weeks and tumor volume (V) calculations were made using the standard formula V= (Width)^2^ x Length/2. At the end of the study (4 weeks), all mice were euthanized by anaesthetizing them using CO_2_ followed by cervical dislocation. For histopathological analysis, tumor samples were fixed in 10% neutral buffered formalin and then processed for paraffin embedding using standard procedures, and 5μM sections of tumor samples were stained with Hematoxylin and Eosin stain. Tumor sections were evaluated microscopically for histopathological changes and representative photomicrographs were captured using Nikon microscope. Animal study was approved by institutional animal care and use committee (IACUC) and all procedures were performed in accordance with the approved protocol and institutional guidelines.

### RNA extraction and quantitative real-time PCR (qRT-PCR)

Total RNA was extracted from control and treatment group HK-2 cells using Trizol reagent and quantified using Nanodrop Spectrophotometer (Thermo Scientific). Gene expression changes were analyzed by single step qRT-PCR amplifications in MyiQ2 real time PCR detection system (BioRad Laboratories, Hercules, CA) using one-step RT-PCR kit with SYBR green and total RNA (200ng). PCR reactions were performed with reverse transcription at 50°C for 15 min, denaturation and reverse transcriptase inactivation at 95°C for 5 min, followed by 40 cycles (10 seconds each) of denaturation at 95°C and annealing and extension at 60°C for 30 seconds. Melt curve analysis was included to confirm the specificity of PCR products. PCR amplification data of each gene were normalized to Ct value of internal housekeeping gene (GAPDH) from the same sample and the fold-changes in gene expression were calculated by using the delta-delta Ct method [87]. List of forward and reverse primer sequences used for RT-PCR analysis have been given in Table 1.

**Table 1:** A list of genes and their primer sequences used for real-time quantitative PCR

Gene	Forward primer (5’-3’)	Reverse primer (5’-3’)	Size (bp)
*GAPDH*	GGTGGTCTCCTCTGACTTCAACA	GTTGCTGTAGCCAAATTCGTTGT	116
*DNMT1*	GTGGGGGACTGTGTCTCTGT	GAAAGCTGCATGTCCTCACA	115
*DNMT3a*	CCTGAAGCCTCAAGAGCAGT	AGCCAAGTCCCTGACTCTCA	143
*MBD4*	CAGGCAAAATGGCAATACCT	GTTTTTGCCCGAAGCTCGTA	136
*HDAC1*	TGGAAATCTATCGCCCTCAC	TCTCTGCATCTGCTTGCTGT	128
*HAT1*	GGTGATTCGTCCTTCCTCA	GCCAGTTTCTTCTCCACTGC	112
*HMT1*	TCCTCGTGCTGTGTGAAGAC	AAACGGTGAGAGATGCTGCT	127

### Western blot analysis

Total cellular protein lysates of HK-2 cells from different treatment and control groups were prepared using RIPA lysis buffer and quantified by Bradford assay. Protein samples (30μg) were separated by gel electrophoresis on a 10% SDS-PAGE, transferred onto nitrocellulose membrane and nonspecific binding sites were blocked by 5% non-fat dried milk solution (containing 0.05% Tween 20) at 4°C. The nitrocellulose membrane was incubated with primary antibody at room temperature for 1 hour. Based on the reaction efficiency, the following dilutions of primary antibodies were used: 1:250 of GAPDH (Santa Cruz, Cat # SC-25778), 1:100 of DNMT1 (Santa Cruz, Cat#10219), 1:100 of HDAC1 (Santa Cruz, Cat# sc-6298), 1:1000 of H3K9Ac (Cell signaling, Cat #9671), 1:1000 of H3K18Ac (Cell signaling, Cat #9441), 1:1000 of H3K27Ac (Cell signaling, Cat #8173), 1: 500 of H3K14Ac, (Cell signaling, Cat #9677) 1:500 of H3K4me (Millipore Cat # 04-745), 1:200 of H3K27me3 (Millipore, Cat#17-622), and 1: 500 of phospho H2AX (Cell signaling, Cat #2577). After giving three washes with washing buffer (1X PBS containing 0.05% Tween 20) for 10 minutes each wash, the membranes were incubated with appropriate secondary antibody (1:1000) conjugated with horseradish peroxidase for 1 h at room temperature. Membranes were washed again with washing buffer for three times (10 minutes each) and signal intensity was captured using an enhanced chemiluminescence detection system (Amersham, NJ). The intensity of protein bands was quantified (using Image J software) and normalized to expression of GAPDH.

### Statistical analysis

To determine the statistical significance of the changes observed in multiple parameters, a two-tailed t-test with paired samples for means (hypothesized difference of 0) was performed. In addition, an analysis of variance (ANOVA) was performed to determine whether the source of variation in the data was between or within treatment groups. The level of significance (α) was set at 0.05 for all statistical tests and data with p value of <0.05 were considered as statistically significant.
